# Biodegradable Poly(Butylene Adipate-Co-Terephthalate) and Thermoplastic Starch-Blended TiO_2_ Nanocomposite Blown Films as Functional Active Packaging of Fresh Fruit

**DOI:** 10.3390/polym13234192

**Published:** 2021-11-30

**Authors:** Danaya Phothisarattana, Phanwipa Wongphan, Khwanchat Promhuad, Juthathip Promsorn, Nathdanai Harnkarnsujarit

**Affiliations:** 1 Department of Packaging and Materials Technology, Faculty of Agro-Industry, Kasetsart University, 50 Ngam Wong Wan Rd., Bangkok 10900, Thailand; danaya.phot@ku.th (D.P.); phanwipa.w@ku.th (P.W.); khwanchat.pro@ku.th (K.P.); juthathip.pro@ku.th (J.P.); 2 Center for Advanced Studies for Agriculture and Food, Kasetsart University, 50 Ngam Wong Wan Rd., Bangkok 10900, Thailand

**Keywords:** food packaging, active packaging, biodegradable, bioplastic, advanced material, fruit, oxygen scavenging, titanium dioxide

## Abstract

Biodegradable polymers can be used for eco-friendly, functional, active packaging to preserve food quality. Incorporation of titanium dioxide (TiO_2_) nanoparticles into polymer packaging enhances ethylene-scavenging activity and extends the shelf-life of fresh produce. In this study, TiO_2_ nanoparticles were incorporated into biodegradable poly(butylene adipate-co-terephthalate) (PBAT)- and thermoplastic cassava starch (TPS)-blended films to produce nanocomposite packaging via blown-film extrusion. The effects of TiO_2_ on morphology, packaging properties, and applications as functional packaging for fresh produce were investigated. Increased TiO_2_ in the film packaging increased amorphous starch content and hydrogen bonding by interacting with the TPS phase of the polymer blend, with negligible chemical interaction with the PBAT component and identical mechanical relaxation in the PBAT phase. Surface topography indicated void space due to non-homogeneous dispersion causing increased oxygen and carbon dioxide permeability. Homogeneous dispersion of fine TiO_2_ nanoparticles increased mechanical strength and reduced oxygen, carbon dioxide, and water vapor permeability. Films containing TiO_2_ also showed efficient oxygen-scavenging activity that removed residual oxygen from the package headspace dependent on the levels and morphology of nanoparticles in the film matrices. Banana fruit packaged in films containing TiO_2_ recorded slower darkening color change and enhanced shelf-life with increasing TiO_2_ content.

## 1. Introduction

Biodegradable plastic materials are promising polymers that are of interest to global packaging manufacturers. Increasing concern about the detrimental environmental impact of plastic packaging drives research and development for alternative biodegradable food packaging. Several biodegradable polymers derived from bioresources and fossil-based synthetic materials including poly(lactic acid) (PLA), poly(butylene adipate-co-terephthalate) (PBAT), poly(butylene succinate) (PBS), starch, and cellulose have been developed into various forms for packaging applications [1,2,3,4]. However, biodegradable polymers have major drawbacks including low barrier properties, low stability during processing and storage, and high water sensitivity [2,5]. These factors limit the utilization of biodegradable plastic as food packaging. Technology that can enhance the properties of biodegradable polymers, particularly as food packaging, is urgently required.

Thermoplastic starch (TPS) is derived from plasticization of starch from several resources e.g., cassava, corn, and potato. It is well known that plant origin has a major effect on the morphology, components, and physicochemical properties of starch films [4]. Cassava is an economic crop in several countries including Thailand, China, and Brazil. Cassava has high processing efficiency and can be readily converted into film and other forms of packaging [5,6]. Development of cassava-starch-based TPS provides significant input for the bioplastic and packaging industries. However, TPS is highly sensitive to water and this causes sticking during the film blowing process, resulting in reduced yield and instability. Blending with other biodegradable polymers including PBAT and PLA improves TPS utilization economy. PBAT is a highly flexible material and can be blended with TPS to improve processability and achieve high stability [5,7,8].

Food security with adequate and appropriate distribution of safe foods has now become a global issue. The development of functional polymers provides extra benefits for packaged products through extended shelf-life [9,10,11,12,13]. Fresh produce typically has a short shelf-life and spoils quickly due to respiration and release of ethylene that accelerates ripening [14]. Attempts to reduce oxygen levels and limit respiration, as well as ethylene scavenging to extend fruit storage life and reduce postharvest loss, have been extensively researched [14,15,16]. 

Titanium dioxide (TiO_2_) has been investigated as a photocatalyst to degrade ethylene and delay the fruit ripening process. TiO_2_ is an inert, non-toxic, and inexpensive substance, with UV-shielding efficiency that can protect light-sensitive packaged goods. The excited state or UV-activated TiO_2_ molecules react with water and oxygen, generating hydroxyl radicals (OH•) and reactive oxygen species that effectively inhibit growth of microorganisms and degrade ethylene compounds [14,15,17,18]. Incorporation of TiO_2_ into films has been mainly investigated in non-biodegradable plastics, while only few investigations have addressed biodegradable polymers. Previous studies produced films by the solution-casting method using solvent as the dispersion medium [19,20,21,22]. Production of blown films by extrusion is more practical for commercial-scale film production. The extrusion process is solventless, and structural formation of the film matrices deviates from solution casting [1,5]. Several researchers indicated that the dispersion of such metal oxide particles influenced the morphology and properties of the films including the mechanical and barrier properties and surface hydrophobicity [19,20,21,22,23]. Metal oxide micro and nanoparticles typically spontaneously agglomerate depending on loading levels and affinity with polymer matrices, giving undesirable dispersion and film properties [23]. Therefore, here, levels of incorporated TiO_2_ were optimized and the effects on film morphology and properties were studied.

This research investigated the effects of TiO_2_ on the morphology and properties of PBAT/TPS-blended films and the shelf-life extension of fresh produce food packaging. Incorporation of TiO_2_ in the films was hypothesized to impact the mechanical properties and permeability while extending the shelf-life of fresh produce. Cavendish bananas were used as a food model. 

## 2. Materials and Methods

### 2.1. Compounding TPS with Titanium Dioxide and PBAT

Acetylated cassava-starch powder (degree of substitution of 0.01–0.03, SMS Corp., Pathum Thani, Thailand) was dried at 50 °C overnight before compounding. Starch, glycerol (C_3_H_8_O_3_, Sac Sci-Eng Ltd., Bangkok, Thailand) and titanium dioxide (TiO_2_) powder (10–40 nm, Rutile:Anatase 85:15, Prime Nanotechnology Co., Ltd., Bangkok, Thailand) were mixed (100:35:0, 100:35:2.5, 100:35:5, 100:35:7.5, 100:35:10, and 100:35:12.5% *w*/*w*) in a dough mixer (SC-236A, Stelang Electric Appliance Co., Ltd, Foshan, China) for 10 min and immediately transferred for compounding in a twin-screw extruder (Labtech Engineering, Muang, Samut Prakan, Thailand). Mixtures were manually fed into the hopper and the heating profile was maintained between 85 °C and 150 °C at 180 rpm screw speed. TiO_2_-compounded TPS (TPS–TiO_2_) extrudates were cut into 2.5 cm pellets using a pelletizer (Labtech Engineering, Muang, Samut Prakan, Thailand). The TPS–TiO_2_ pellets were manually blended with PBAT pellets at a PBAT/TPS ratio of 60/40. The blended pellets were compounded in the twin-screw extruder with temperatures ranging 80 °C to 145 °C at 180 rpm screw speed. The compound was pelletized into 2.5 cm pellets.

The melt flow index (MFI) of PBAT/TPS with titanium dioxide pellets was determined. The pellets were dried overnight at 50 °C before testing. The MFI of 5 g pellets was determined using ASTM1238-10 by multi-weight melt-flow tester (MF30, Instron, Norwood, MA, USA) at 190 °C and 2.16 kg load cell. Triplicate samples were calculated for MFI and reported in g/10 min.

### 2.2. Blown-Film Extrusion

The blended pellets were blown using a single-screw blown-film extruder (Labtech Engineering, Samut Prakan, Thailand) at a temperature range between 150 °C and 165 °C in the barrel and 165 °C for the die temperature. Nip-roll and screw speeds were 2.1–3.2 rpm and 20–27 rpm, respectively. The air flow was adjusted to form stable vertical tubular bubbles during blown-film extrusion. Films were collected and stored in aluminum bags until further measurement. 

### 2.3. Morphology

#### 2.3.1. Fourier-Transform Infrared Spectroscopy (FTIR)

Infrared absorption spectra of 1 cm × 1 cm films were determined using a Bruker Tensor 27-FT-IR spectrometer (Bruker OPTIK GmbH, Leipzig, Germany) in attenuated total reflectance (ATR) mode and diamond tip (45° anvil geometry). FTIR spectra were collected at wavenumbers between 500 and 4000 cm^−1^ at 4 cm^−1^ resolution with 64 scanning times. Intensity ratios were calculated from two replications.

#### 2.3.2. Scanning Electron Microscopy (SEM)

The films were dipped in liquid nitrogen for freeze crack, and fixed on a metal stub with adhesive tape. Specimens were coated with gold using a sputter coater (Quorum Technology Polaron Range SC7620, East Sussex, UK). Microstructures were determined by a scanning electron microscope (SEM, FEI Quanta 450, Thermo Fisher Scientific, Waltham, MA, USA) at 10 kV and magnification of 1000× and 5000× for surface and cross-section area, respectively.

#### 2.3.3. Atomic Force Microscopy (AFM)

Topographic images 5 μm × 5 μm size of the film surfaces were determined by atomic force microscopy (AFM, MFP-3D-Bio, Asylum Research, Oxford Instruments Santa Barbara, CA, USA). Tapping mode at 225 μm-length cantilever was performed at 190 kHz resonance frequency, 8 nm nominal tip radius curvature and 0.8 Hz scanning rate [24].

### 2.4. Dynamic Mechanical Thermal Analysis (DMTA)

Thermomechanical properties of the films were determined using a dynamic mechanical thermal analyzer (DMTA, Mettler Toledo, Greifensee, Switzerland). Films were analyzed in tension mode between −100 and 100 °C at 2 °C/min scanning rate and frequencies of 0.5, 1.0, and 5.0 Hz. Relaxation temperature was determined by tan δ.

### 2.5. Mechanical Properties

Tensile strength (TS), elongation at break (EB), and Young’s modulus (YM) were measured following ASTM D882-12 using an Instron universal testing machine (Model 5965, Instron, Norwood, MA, USA) in machine direction (MD) and cross direction (CD). Thickness of the films (2.5 cm × 10 cm) was measured in five replications using a digital micrometer and conditioned in a humidity chamber at 50% RH at 25 °C for 48 h before testing. Films (six replicates) were placed between the grips at a distance of 5 cm. The samples were measured for mechanical strength using a rate of 500 mm/min for crosshead speed.

### 2.6. Barrier Properties

#### 2.6.1. Water Vapor Permeability

Films were cut into 7 cm diameter circles and determined for water vapor transmission rate (WVTR) using the standard cup method (ASTM E96). Triplicate films were placed on aluminum cups containing dehydrated silica gel and covered with an O-ring before sealing with molten paraffin. Samples were stored in a humidity chamber (25 °C and 50% RH) and weighed daily until the weight was constant. WVTR was calculated from the linear slope of the plot between weight gain and storage time. Water vapor permeability (WVP) was calculated using Equation (1).

WVP = (WVTR × L)/ΔP(1)

where L and ΔP are film thickness (mm) and difference in water vapor partial pressure (atm) between the two sides of the film, respectively.

#### 2.6.2. Oxygen Permeability

Oxygen transmission rate (OTR) was determined following ASTM D3985-051 (ASTM, 2010) using an 8500 model oxygen permeability analyzer (Illinois Instruments, Inc., Johnsburg, IL, USA) at 23 ± 2 °C and 50% RH. Films were measured in triplicate (13 cm diameter circles) and calculated for oxygen permeability (OP) following Equation (2).

OP = (OTR × L)/ΔP(2)

where L and ΔP are the thickness (mm) and oxygen partial pressure difference (atm) between the two sides of the film, respectively.

#### 2.6.3. Carbon Dioxide Permeability

Carbon dioxide transmission rate (CO2TR) was determined by a carbon dioxide permeability analyzer (PERMATRAN-C, Model 4/41 Module MCT, USA) at 23 °C and 0% RH. Films were measured in two replications and calculated for oxygen permeability (CO2P) following Equation (3).

CO_2_P = (CO_2_TR × L)/ΔP (3)

where L and ΔP are the thickness (mm) and carbon dioxide partial pressure difference (atm) between the two sides of the film, respectively.

#### 2.6.4. Light Transmission

Light transmission was determined using a UV-vis spectrophotometer (Evolution 300, Thermo Scientific, BEC Thai Bangkok Equipment & Chemical Co., LTD., Bangkok, Thailand) at wavelengths between 200 and 800 nm. Triplicate films (3 cm × 4 cm) were attached between the metal plates with a slit for light transmission. 

### 2.7. Oxygen-Scavenging Capacity

Reduction of oxygen concentration in package headspace was determined using a headspace gas analyzer (Dansensor^®^ Checkmate 3, Dansensor, Ringsted, Denmark). The PBAT/TPS films (12.5 cm × 10 cm) were placed in a 130 mL plastic tray (approximately 6 cm × 10 cm × 2.1 cm dimensions) and stored in high-barrier nylon bags (OPA/LLDPE) before heat sealing. Triplicate packages were stored in ambient conditions and exposed to laboratory light and sunlight during the daytime. Septa were adhered to each package at the beginning of the test. Gas components were collected using a needle connecting with the headspace analyzer through the septum. The oxygen-scavenging capacity was calculated from Equation (4).

Oxygen-scavenging capacity (%) = (C_0_ − C_t_/C_0_) ×100 (4)

where C_0_ and C_t_ are the concentrations of oxygen at initial (t = 0) and storage time, respectively.

### 2.8. Application as Banana Packaging

Films were exposed under UV lamps for 7 min before sealing on three sides using an impulse bar sealer. Cavendish bananas were bought from a local market in Bangkok, Thailand. Individual bananas were packed in 10 cm × 20 cm bags produced from PBAT/TPS with TiO_2_ contents 0, 3, and 5% (*w*/*w*). Samples were stored in ambient condition (25 °C, laboratory light, and natural daylight). Appearances of the bananas were recorded during storage for 12 days.

### 2.9. Statistical Analysis

Samples were determined for significant differences using analysis of variance (ANOVA). Data analyses were performed using Duncan’s multiple range test with 95% confidence intervals using SPSS 17.0 (SPSS Inc., Chicago, IL, USA).

## 3. Results and Discussion

### 3.1. Surface Chemistry

**Figure 1:**
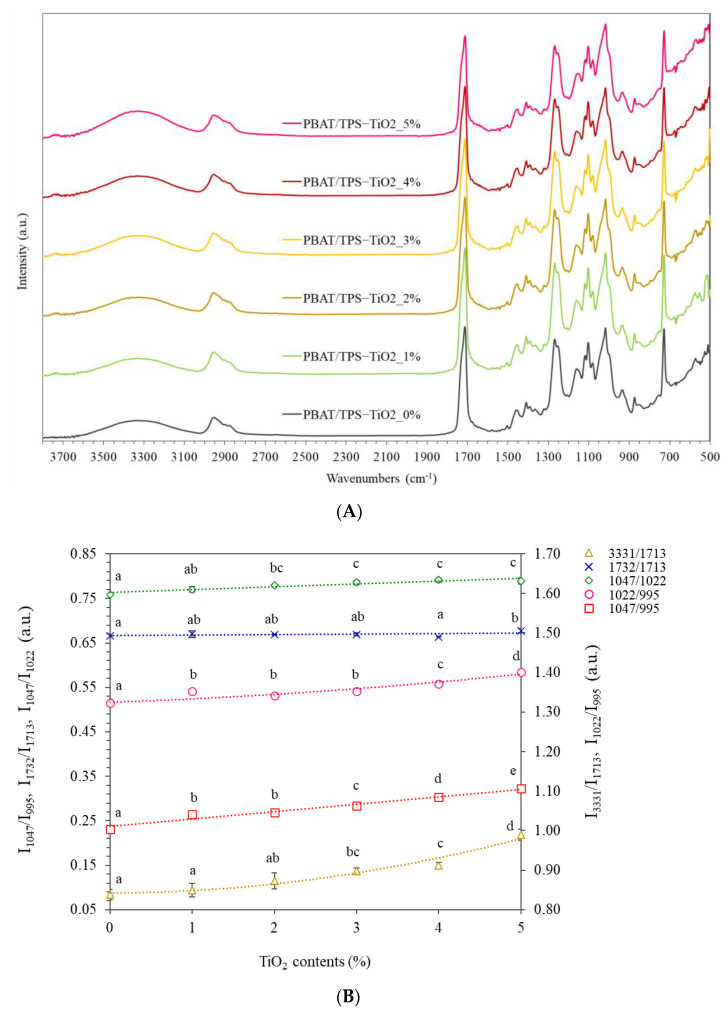
Surface chemistry shown by (**A**) FTIR absorption spectra and (**B**) intensity ratios of the selected absorption peaks of PBAT- and TPS-blended films (PBAT/TPS) containing different contents of TiO_2_ (1, 2, 3, 4, and 5%). The different letters (a–e) indicate significant difference (*p* ≤ 0.05) between samples (as different concentrations of TiO_2_).

Blown films consisted of PBAT and TPS blended with TiO_2_ at concentrations between 1% and 5%. IR-absorption spectra and intensity ratios of the blended films are shown in Figure 1A,B. The PBAT/TPS films with different TiO_2_ contents showed typical IR absorption peaks between 500 and 1500 cm^−1^ (fingerprint region), attributed to PBAT and TPS components (Figure 1A). PBAT showed major IR absorption peaks at 728, 874 cm^−1^ (out-of-plane bending), and 1018 cm^−1^ (in-plane bending) ascribed to the bending vibration of phenyl ring structures, while C–O aromatic esters and C=O carbonyls caused peaks at 1270 and 1714 cm^−1^, respectively [1,2]. Incorporation of TiO_2_ at up to 5% gave identical locations of the absorption peaks, suggesting that TiO_2_ had no effects on chemical bonding in PBAT polymers.

TPS showed absorption peaks located at 1018 and 1080 cm^−1^ assigned to C–O stretching vibration of the C–O–C group in the glucose unit [1]. The peak at 1018 cm^−1^ merged with shoulders at 1047 and 995 cm^−1^ that were sensitive to the degree of crystallinity and amorphous starch, respectively. Intensity ratios between these peaks were used to quantify the degree of order structures in starch [4]. The wide peak location between 3000 and 3650 cm^−1^ (centering at 3330 cm^−1^) was attributed to O–H stretching vibration due to inter- and intra-molecular hydrogen bonding of the films. Hydrogen bonding in PBAT/TPS-blended films was mainly attributed to hydrogen bonding of the hydroxyl groups in starch polymers. Incorporation of TiO_2_ increased the intensity of the bands, suggesting an increased degree of hydrogen bonding. Oxygen atoms in TiO_2_ formed hydrogen bonding with H atoms in the polymers. TiO_2_ also showed a small absorption peak at 3754 cm^−1^, also reported by Mohamed et al. [18], due to free hydroxyl groups located on TiO_2_ at different crystallographic planes and Ti^4+^–O^2−^ pairs.

The major peak at 1714 cm^−1^ merged with a shoulder at 1731 cm^−1^ and was attributed to the crystalline and amorphous structures of PBAT, respectively [2,25]. The intensity ratios between these aforementioned peaks were identical in all films (0–5% TiO_2_), indicating that TiO_2_ had no effect on the crystallinity of PBAT components. Conversely, the intensity of TPS absorption was clearly modified depending on TiO_2_ contents (Figure 1B). The absorption peak at 1022 cm^−1^ was sensitive to amorphous structures, while peak intensities at 995 and 1047 cm^−1^ were dependent on the degree of crystalline structures of TPS [3]. The intensity ratios between peaks at 1022 and 995 cm^−1^ (I1022/I995) increased as TiO_2_ increased, giving values of 1.33 and 1.40 for 0% and 5% TiO_2_, respectively. Increasing I1022/I995 indicated higher amounts of amorphous TPS. Results suggested that incorporation of TiO_2_ enhanced melting of the polymers. Melt-flow index (MFI) of the PBAT/TPS pellets decreased with addition of TiO_2_ (2.4–3.4 g/10 min) compared with the control (4.4 g/10 min). Lower MFI was related to higher melt viscosity that facilitated heat transfer and polymer melting in extrusion [26]. Ostafińska et al. [21] reported increased shear moduli values with added 3% TiO_2_, while TiO_2_ also caused degradation of the TPS polymer at elevated temperature, forming a higher amorphous fraction of starch. A third-order polynomial equation fitted well to increased I1022/I995 as a function of TiO_2_ content. Diverse correlation was found for 1% TiO_2_-containing films that had higher values, indicating higher fractions of amorphous TPS. Different FTIR results were also coincident with mechanical properties, as discussed later. 

The intensity ratio between peaks at 1047 and 995 cm^−1^ (I1047/I995), attributed to crystalline structures of TPS, significantly increased from 1.0 to 1.1 for 0% and 5% TiO_2_ (Figure 1B), while alteration of C–C bond vibrations reflected the modified crystalline structures of the TPS phase due to TiO_2_. The intensity of the peak at 3330 cm^−1^ also increased with TiO_2_ content, suggesting the formation of H-bonding. Similarly, Oleyaei et al. [20] indicated hydrogen bonding between starch and TiO_2_ in potato starch films produced by solution casting, reflected by shifting of the O–H stretching peak. Results suggested formation of hydrogen bonding between oxygen atoms in TiO_2_ and hydrogen atoms in starch chains that altered the morphology of starch crystallites. 

### 3.2. Microstructures

**Figure 2:**
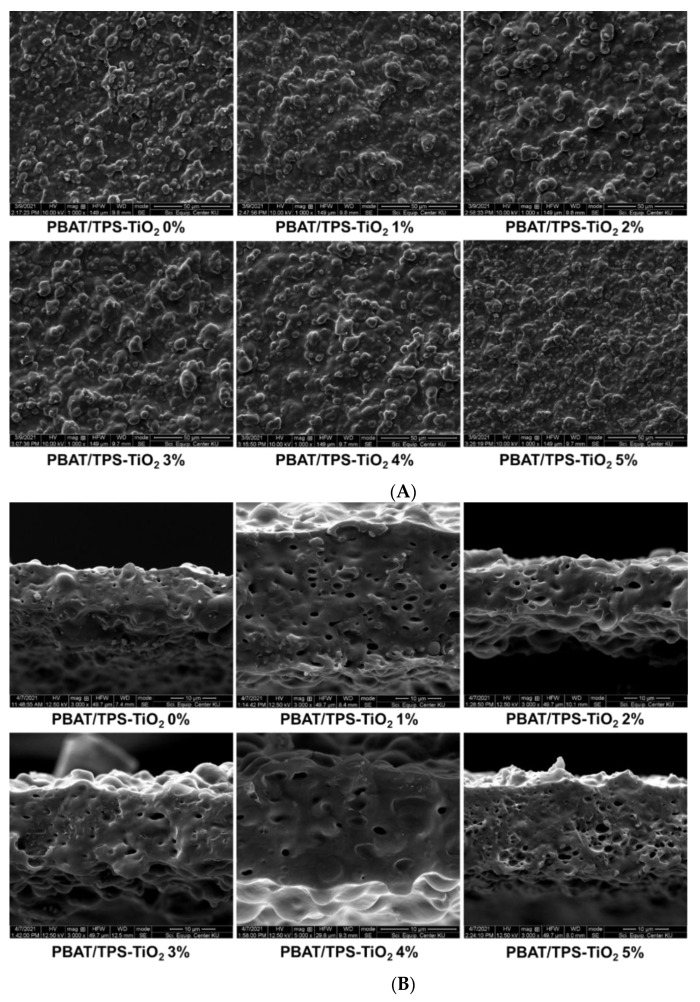
Microstructures as (**A**) surface and (**B**) cross-section of PBAT- and TPS-blended films (PBAT/TPS) containing different contents of TiO_2_ (1, 2, 3, 4, and 5%).

Microstructures of the PBAT/TPS blown films containing different TiO_2_ contents (1% to 5%) are shown in Figure 2. All films had a rough surface with numerous fine granules (approximately 5–20 μm diameter) dispersed in the matrices (Figure 2A). TPS consisted of high numbers of hydrophilic hydroxyl groups and was highly hydrophilic, while PBAT mainly consisted of hydrophobic hydrocarbons with benzene rings, giving immiscible polymer blends. Incompatibility between PBAT and TPS caused phase separation in the microscale of the films [5,7,14,27]. Incomplete melting of the polymers also caused fine granule embedding on the film surface. Films with no TiO_2_ (control) had the finest size of dispersed granules. Addition of TiO_2_ merged the granules, forming clumps with larger size. Increased TiO_2_ from 1% to 2% gave larger particles, while PBAT/TPS with TiO_2_ at 2% to 4% had similar surface structure. Conversely, 5% TiO_2_ gave smaller dispersed granules, similar to the control film. Increasing TiO_2_ increased the amorphous phase of TPS (Figure 1B), with improved dispersion of starch networks in continuous PBAT matrices (compared with starch crystallites), while the degree of hydrogen bonding sharply increased in 5% TiO_2_ and favorably interacted with TPS. A higher degree of starch melting and amorphous structures facilitated dispersion of TiO_2_ in the blend films [21], giving finer particle size in 5% TiO_2_ films. Accordingly, higher interaction between TiO_2_ and polymer matrices enhanced particle dispersion, with reduced tendency for particle aggregation [23].

Cross-section microstructures are shown in Figure 2B. Incorporation of TiO_2_ caused higher numbers of larger pores embedding in the film matrices. The cross-sections also revealed dispersion of intact (non-melted) polymer granules underneath the surface of the film matrices, causing non-smooth structures on both surface and cross-section. Goudarzi et al. [17] similarly found rough structures of dispersed TiO_2_ nanoparticles in cross-sectional wheat starch films due to the high surface energy of the nanoparticles. PBAT/TPS–TiO_2_ 0% and 5% showed smoother cross-sections due to the smaller size of dispersed granules, as shown in Figure 2A. Results indicated that addition of TiO_2_ from 1% to 4% formed larger clumps, while TiO_2_ at 5% was smoother due to smaller granule size. 

### 3.3. Surface Topography

**Figure 3:**
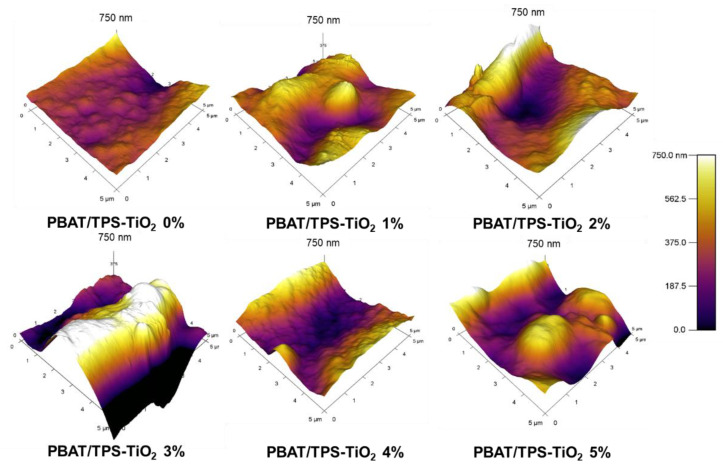
Surface topographic images of PBAT- and TPS-blended films (PBAT/TPS) containing different contents of TiO_2_ (1, 2, 3, 4, and 5%).

AFM topographic images showed the surface morphology of the films (Figure 3). Peak and valley characteristics reflected surface roughness and were strongly dependent on concentrations of TiO_2_. Lighter areas (white color) indicated the height of the surface, while darker areas (black color) represented deep valleys and suggested possible formation of micropores. PBAT/TPS films with no TiO_2_ had the smoothest surface morphology. Increasing TiO_2_ content increased surface roughness, contributing to higher peaks and deeper valleys. PBAT/TPS–TiO_2_ 3% gave the largest deviation of surface height i.e., larger area and higher intensity of light and dark color. Void spaces typically formed in non-compatible materials that caused increased permeation of volatile substances [24]. These surface peaks and valleys affected the permeability of the films, as discussed later. Oleyaei et al. [20] also indicated that addition of TiO_2_ increased surface roughness and reduced the uniformity of starch films by forming aggregates. Addition of TiO_2_ at 4% and 5% reduced the deep valleys and deviations of surface height. TiO_2_ nanoparticles at higher concentrations filled the pores and void spaces, reducing roughness [19]. Moreover, the AFM image of PBAT/TPS–TiO_2_ 5% also revealed distinct fine particles of 1–2 μm, coincident with the SEM images. 

### 3.4. Mechanical Relaxation

**Figure 4:**
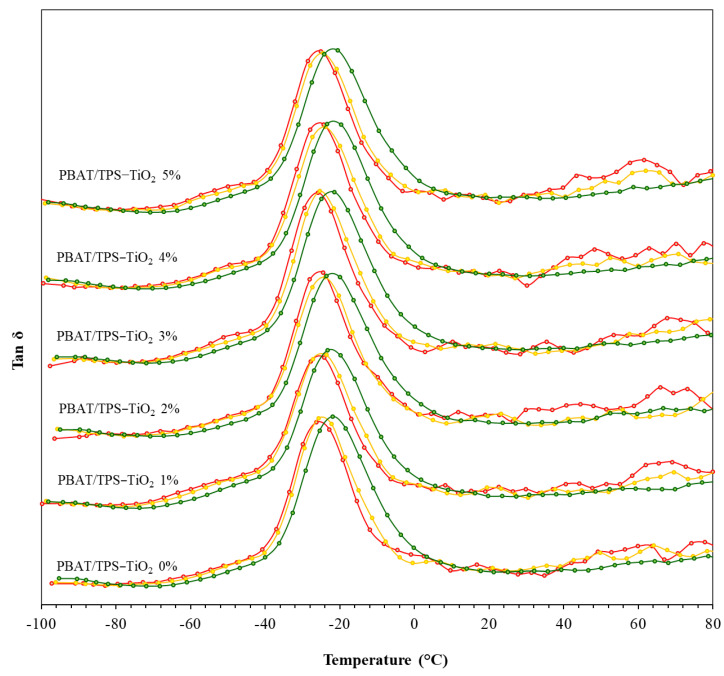
DMTA of PBAT- and TPS-blended films (PBAT/TPS) containing different contents of TiO_2_ (1, 2, 3, 4, and 5%) at different frequencies of 0.5 Hz (red), 1.0 Hz (yellow), and 5.0 Hz (green).

Figure 4 shows tan δ derived from the ratio between storage modulus and loss modulus. The peak of tan δ was attributed to phase transition of the film components, reflecting the glass transition phenomenon in the amorphous matrices. Glass transition and relaxation are important parameters determining the state and molecular mobility of amorphous polymers that affect mechanical properties and stability of biomaterials. Above glass-transition temperature (*T*_g_), molecular mobility increased causing a sharp increase in tan δ, concurrent with a sharp drop in the storage modulus [20,28,29]. The tan δ peak was frequency-dependent and, therefore, considered as mechanical α-relaxation. The peak temperature indicated α-relaxation temperature (*T*α). A small shoulder at low temperature (from −75 °C to 40 °C) was due to the glycerol-rich phase of the TPS components [14]. The sharp peak between −40 °C and 10 °C was due to PBAT components, corresponding with Tα between −25 °C (0.5 Hz) and −20 °C (5 Hz). All films showed identical peak location and intensity, suggesting that TiO_2_ concentration had no effect on magnitude or temperature of the α-relaxation in the PBAT phase. However, a slightly sharper intensity of tan δ above 25 °C occurred with increasing TiO_2_ (particularly at higher frequency). Interaction between TiO_2_ and TPS via H-bonding (as suggested by FTIR) possibly enhanced the magnitude of the mechanical relaxation. Monfared and Jamshidi [23] indicated that chemical interaction between TiO_2_ particles and polymer matrices increased DMTA modulus intensity. Results indicated that TiO_2_ influenced mechanical behavior, involving molecular mobility of the TPS phase, while interaction with PBAT was negligible.

### 3.5. Mechanical Properties

**Figure 5:**
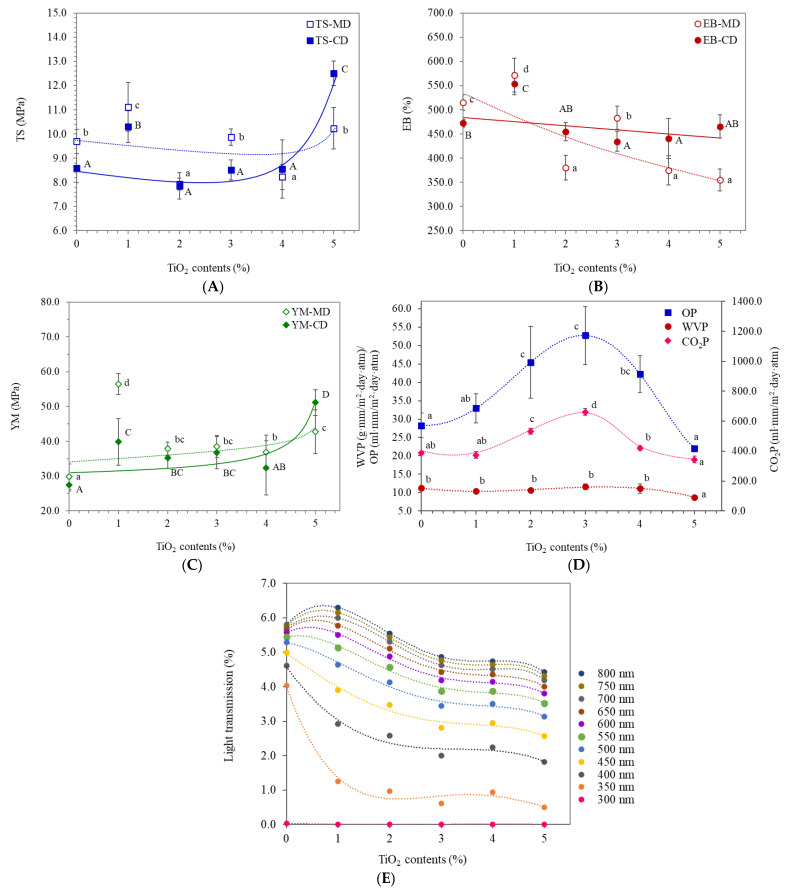
Properties of PBAT- and TPS-blended films (PBAT/TPS) containing different contents of TiO_2_ (1, 2, 3, 4, and 5%) as a function of TiO_2_ contents namely (**A**) tensile strength, (**B**) elongation at break, (**C**) Young’s modulus, (**D**) water vapor permeability (WVP), oxygen permeability (OP), and carbon dioxide permeability (CO_2_P), and (**E**) light transmission (CD and MD indicate cross direction and machine direction of the films in the blowing process, respectively). The different letters (a–c and A–C) indicate significant difference (*p* ≤ 0.05) between samples (as different concentrations of TiO_2_).

Tensile strength (TS), elongation at break (EB), and Young’s modulus (YM) of the films in the machine direction (MD) and cross-direction (CD) are shown in Figure 5A–C. A sharp increase in TS was recorded with 1% TiO_2_ in both MD and CD (Figure 5A). FTIR revealed a high amorphous fraction of TPS in films containing 1% TiO_2_. The PBAT/TPS ratio was 60/40 and, therefore, TPS possibly acted as a dispersed phase in continuous PBAT. Formation of amorphous TPS networks increased the dispersion of TPS molecules in PBAT matrices. Further increased TiO_2_ formed non-homogeneous matrices. TiO_2_ particles inhibited connection and adhesion between polymer networks, resulting in lower TS values. Goudarzi et al. [17] also reported insignificant change of TS in starch and TiO_2_ nanocomposites up to 5% TiO_2_. The TS-CD sharply increased with increasing TiO_2_ above 4%, suggesting reinforcement of TiO_2_ in polymer matrices. Such increasing TS corresponded with finer particles dispersed in film matrices, as determined by SEM (Figure 2). Oleyaei et al. [20] showed increased TS and decreased EB of potato starch films with well-dispersed TiO_2_, while agglomerated nanoparticles discontinued the mechanically equilibrated film system that consequently reduced TS and EB values. 

The EB of the films decreased as TiO_2_ increased due to non-homogeneous film matrices (Figure 5B). Adhesion between polymer networks increased the ability to extend without rupture. Homogeneous distribution and volume fraction of dispersed fillers in the films, and their interaction with networks strongly influenced elongation [20,30,31,32]. Dispersion of TiO_2_ and interaction with starch molecules reduced adhesion force between the polymer chains. EB sharply decreased in MD, with TiO_2_ at 2, 4, and 5%. TiO_2_ at 1% gave the highest EB values, suggesting fine dispersion of TiO_2_, particularly in TPS matrices. YM increased with incorporation of TiO_2_, particularly at 1% (Figure 5C). Further increase in TiO_2_ had insignificant effects, while TiO_2_ at 5% had higher YM. Poor dispersion of TiO_2_ fillers at 2% to 4% gave lower stiffness of PBAT/TPS films [21,33], while the effect of TiO_2_ content on YM was similar to the TS values. 

### 3.6. Barrier Properties

Permeation of volatile substances, including water vapor and gas, through film matrices directly influences the quality and shelf-life of packaged food and agriculture. Water vapor permeability (WVP), oxygen permeability (OP), and carbon dioxide permeability (CO_2_P) are shown in Figure 5D. OP and CO_2_P increased with increasing TiO_2_, reaching maximum permeability at 3% TiO_2_. The AFM images revealed the largest and deepest area of voids in PBAT/TPS films containing 3% TiO_2_, corresponding with the highest gas permeability (OP and CO_2_P). Micro-voids allowed higher mass transfer and, therefore, gas diffusion through polymer matrices that increased permeability. The diatomic oxygen molecule in O_2_ causes the non-polar characteristic of O_2_ gas. Carbon dioxide (CO_2_) consists of symmetric polar bonding between C and O; however, the molecule is linear causing no net molecular dipole moment and, therefore, is non-polar. O_2_ and CO_2_ are both non-polar molecules with similar permeation behavior. Further increase in TiO_2_ to 5% decreased OP and CO_2_P. The AFM images also showed that TiO_2_ at 4% and 5% gave less diversion of surface height with reduced void area, concurrent with reduced gas permeability.

Water vapor is a hydrophilic substance that readily transfers through hydrophilic TPS matrices. The FTIR data revealed that increasing TiO_2_ led to increasing the amorphous TPS phase with increased permeation of water vapor. However, WVP values of the films were insignificantly different with increasing TiO_2_ up to 4% (Figure 5D). Moreover, the void space formed on the surface showed insignificant effects on WVP values that ranged from 10.4 to 11.6 g·mm/m^2^·day·atm. Oleyaei et al. [20] also indicated insignificant reduction of WVP in films with non-homogeneous TiO_2_ dispersion and aggregates. FTIR and DMTA indicated no interaction between TiO_2_ and PBAT and no effect of TiO_2_ on crystallinity of the PBAT phase. Conversely, TiO_2_ modified the morphology and amorphous phase of TPS. Insignificant changes of WVP suggested a major role of PBAT as a continuous hydrophobic phase (PBAT/TPS 60/40) that limited water vapor diffusion. TiO_2_ at 5% reduced the WVP of PBAT/TPS to 8.8 g·mm/m^2^·day·atm, coincident with the finest dispersed granules, as shown by SEM. The finely dispersed nanoparticles formed a tortuous pathway for the transfer of water vapor and volatile molecules through the matrices, limiting diffusion and permeation, while hydrogen bonding between Ti and O and hydroxyl groups in starch decreased WVP [17,20]. Results indicated that the homogeneous dispersion of TiO_2_ particles was a major factor decreasing permeability.

Light transmission of films influences the quality deterioration of light-sensitive products including color, vitamins, and oxidative degradation. Figure 5E shows light transmission of the films as a function of TiO_2_ contents at different wavelengths. Incorporation of 1% TiO_2_ sharply decreased light transmission of the films, particularly in the UV range that had higher energy and accelerated quality loss of packaged products. Goudarzi et al. [17] demonstrated high UV protective properties of TiO_2_-incorporated wheat starch films that increased with increasing TiO_2_.

A slight increase in light transmission above 650 nm was found in PBAT/TPS films containing 1% TiO_2_, coincident with a higher degree of amorphous TPS, while the amount of TiO_2_ that prevented light was low. Further increase in TiO_2_ increased amorphous TPS and light transmission reduced, suggesting the role of TiO_2_ as a light barrier. TiO_2_ at 5% gave the lowest light transmission.

### 3.7. Oxygen-Scavenging Capacity

**Figure 6:**
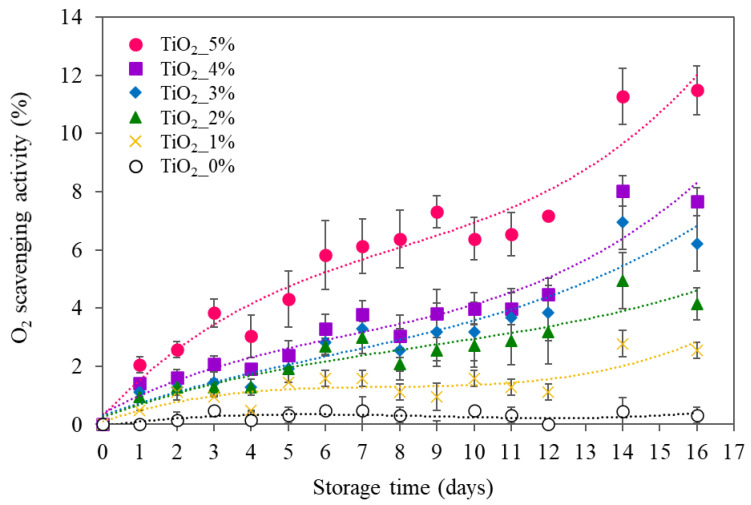
Oxygen-scavenging activity of PBAT- and TPS-blended films (PBAT/TPS) containing different contents of TiO_2_ (1, 2, 3, 4, and 5%) as a function of storage time.

Residual oxygen in food packaging accelerates quality deterioration. Modified-atmosphere packaging and vacuum packing cannot completely remove residual oxygen and small amounts (1% to 5%) of oxygen gas cause quality loss [34]. PBAT/TPS films containing TiO_2_ showed efficacy to reduce O_2_ gas in the package headspace (Figure 6). UV rays activate TiO_2_ to react with oxygen and water, producing several free radicals including O•_2_, HOO•, and HO•. These free radicals readily accelerated photochemical reactions through interaction with starch polymers [15]. Consequently, reactive oxygen species that interacted with polymers were eliminated from the package headspace. 

The degree of oxygen scavenging was strongly dependent on the TiO_2_ content. Higher TiO_2_ gave faster rates and higher levels of oxygen removal. Kordjazi and Ajji [16] also found that increasing TiO_2_ catalyzed oxygen absorption of the hydroxyl-terminated polybutadiene blends with low density polyethylene and polylactic acid blended matrices. Figure 6 shows that the oxygen-scavenging capacity during storage fitted well to a third-order polynomial equation. Films with lower TiO_2_ reached equilibrium oxygen concentration faster than higher TiO_2_. Films containing 2, 3, and 4% had similar scavenging activity, while 5% TiO_2_ showed higher capacity. The SEM images displayed finely dispersed TiO_2_ particles in films containing 5% TiO_2_. Higher numbers of smaller particles gave greater surface area for oxygen-scavenging activity, giving the highest capacity. Conversely, the SEM images showed similar microstructures of films with 2, 3, and 4% TiO_2_ particles namely larger and highly connected oval particles, giving less surface area of TiO_2_. Results indicated that the oxygen-scavenging capacity of films containing TiO_2_ was dependent on TiO_2_ content and microstructure i.e., surface area. 

### 3.8. Application as Banana Packaging

**Figure 7:**
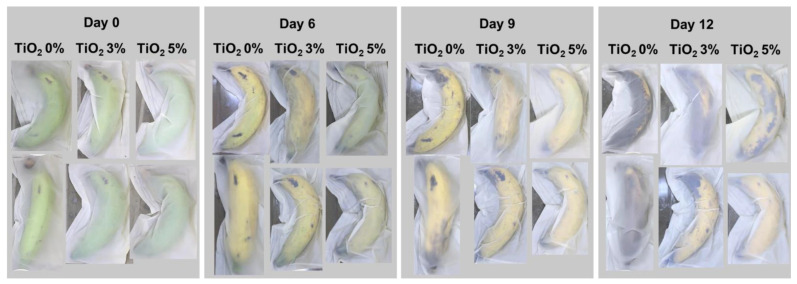
Appearance of banana packaged in PBAT- and TPS-blended films (PBAT/TPS) containing different contents of TiO_2_ (0, 3, and 5%) stored at ambient condition (25 °C, laboratory and natural daylight) for 12 days.

Films were used as banana packaging and stored at ambient condition, as shown in Figure 7. Films with no TiO_2_ (control) exhibited faster color changes and decay. The green banana turned yellow on day 3 in samples packaged in control films. Degree of yellowness decreased with increasing TiO_2_ at 3% and 5%, respectively. TiO_2_ has ethylene scavenging activity under UV light. Ethylene is a plant hormone that releases in the ripening stage of fresh produce causing chlorophyl degradation and reduced fruit firmness. Removal of ethylene from the package headspace can delay fruit ripening [14]. de Matos Fonseca et al. [15] developed TiO_2_ photocatalytic coatings on expanded polyethylene foam nets for ethylene elimination. Exposure of the TiO_2_ nanocrystals with UV light (≤387.5 nm) causes photon (hν) absorption on the surface and excitation of electrons from the valence band (h_vb_^+^) to the conduction band (e_cb_^−^). These reactions produce strong reactive oxygen species (ROS) including hydroperoxyl (HO_2_•) and hydroxyl (HO•) radicals that further react with unsaturated carbon bonds (C=C) in ethylene molecules causing cleavage and degradation [14,15,35,36]. 

The UV-activated oxidizing activity of TiO_2_ slows the process of chlorophyl degradation and, therefore, reduces the incidence of yellowness in banana. On day 9 the banana in the control package showed black spots, indicating consumer rejection, while TiO_2_ at 5% in films gave the best appearance without darkening. The permeability of O_2_ and CO_2_ also influenced respiration of fresh produce and governed the storage life [14,15]. Figure 5D revealed lower OP and CO_2_P in films containing 5% than 3% TiO_2_, while the 5% TiO_2_ films had the highest oxygen-scavenging activity. Reduction of oxygen permeation and concentration in the package by 5% TiO_2_ reduced the respiration of banana, giving better storage life. Accordingly, combined limited respiration and ethylene scavenging capacity in PBAT/TPS–TiO_2_ 5% films gave the highest shelf-life extension capacity. 

## 4. Conclusions

Incorporation of TiO_2_ (1% to 5%) in PBAT/TPS blends via blown-film extrusion extended the shelf-life of fresh produce packaging. PBAT/TPS films containing TiO_2_ had oxygen-scavenging capacity, dependent on both TiO_2_ levels and the surface area for oxygen exposure. The films showed high efficiency in extending the storage life of banana by decreasing the rate of color change (green to yellow) and darkening, with increased storage life of more than three days. Dispersion of TiO_2_ into PBAT/TPS matrices formed hydrogen bonding with starch and modified the morphology of the TPS phase, with minimal effect on PBAT matrices. Moreover, the mechanical properties and permeability of O_2_ and CO_2_ were dependent on the microstructures and void space in the film matrices. PBAT/TPS with TiO_2_ at 5% gave the least permeability with the highest tensile strength, oxygen-scavenging, and shelf-life extension capacity for fresh banana. This investigation showed enhanced functional performance of bioplastic food packaging to extend the shelf-life of fresh produce.

## Data Availability

The data presented in this study are available on request from the corresponding author.
